# Human single-stranded DNA binding protein 1 (hSSB1/NABP2) is required for the stability and repair of stalled replication forks

**DOI:** 10.1093/nar/gku276

**Published:** 2014-05-05

**Authors:** Emma Bolderson, Eva Petermann, Laura Croft, Amila Suraweera, Raj K. Pandita, Tej K. Pandita, Thomas Helleday, Kum Kum Khanna, Derek J. Richard

**Affiliations:** 1 Genome Stability Laboratory, Cancer and Ageing Research Program, Institute of Health and Biomedical Innovation, Translational Research Institute, Queensland University of Technology, Woolloongabba, Queensland, 4102, Australia; 2 School of Cancer Sciences, College of Medical and Dental Sciences, University of Birmingham, Edgbaston, Birmingham B15 2TT, UK; 3 Department of Radiation Oncology, University of Texas Southwestern Medical Center, 5323 Harry Hines Boulevard, Dallas, TX 75390, USA; 4 Science for Life Laboratory, Division of Translational Medicine and Chemical Biology, Department of Medical Biochemistry and Biophysics, Karolinska Institutet, S-171 21 Stockholm, Sweden; 5 Signal Transduction Laboratory, QIMR Berghofer Medical Research Institute, Brisbane, Queensland 4029, Australia

## Abstract

Aberrant DNA replication is a primary cause of mutations that are associated with pathological disorders including cancer. During DNA metabolism, the primary causes of replication fork stalling include secondary DNA structures, highly transcribed regions and damaged DNA. The restart of stalled replication forks is critical for the timely progression of the cell cycle and ultimately for the maintenance of genomic stability. Our previous work has implicated the single-stranded DNA binding protein, hSSB1/NABP2, in the repair of DNA double-strand breaks via homologous recombination. Here, we demonstrate that hSSB1 relocates to hydroxyurea (HU)-damaged replication forks where it is required for ATR and Chk1 activation and recruitment of Mre11 and Rad51. Consequently, hSSB1-depleted cells fail to repair and restart stalled replication forks. hSSB1 deficiency causes accumulation of DNA strand breaks and results in chromosome aberrations observed in mitosis, ultimately resulting in hSSB1 being required for survival to HU and camptothecin. Overall, our findings demonstrate the importance of hSSB1 in maintaining and repairing DNA replication forks and for overall genomic stability.

## INTRODUCTION

The DNA damage response is a crucial component of the surveillance network that maintains the stability and integrity of the genome. In order for genomic integrity to be maintained, faithful DNA replication is essential. It is estimated that the replication of the human genome is initiated at over 50 000 ‘origins of replication’ (1). Consequently, the accurate replication of DNA requires the correct functioning of many replication forks within each round of replication. When the progression of a replication fork is impeded, it is essential that the components associated with the fork are retained to allow the replication fork to restart following fork repair or removal of the blockage. Significantly, defects in the pathways involved in the recovery and stabilisation of stalled replication forks lead to genomic instability and chromosomal rearrangements, both of which are key hallmarks of cancer cells (2). Thus, this highlights the importance of characterizing proteins and pathways involved in repair of stalled replication forks.

DNA replication forks can be stalled by secondary DNA structures, transcription complexes and a number of DNA lesions including chemically modified bases and interstrand crosslinks. DNA obstacles that specifically block the progression of DNA polymerases can lead to uncoupling of the replicative polymerase and helicase activities, with the helicase continuing to generate long stretches of single-stranded DNA (ssDNA) or ssDNA can be generated via resection by nucleases such as Mre11 (3). Replication fork stalling activates the ATR kinase that subsequently phosphorylates other proteins to activate cell cycle checkpoints and DNA repair. An important downstream target of ATR is the checkpoint kinase Chk1, which is phosphorylated and activated following replication fork stalling (4). This checkpoint signalling cascade down-regulates origin firing and activates proteins involved in the stabilisation of the fork. A stalled replication fork can be resolved by a number of mechanisms including removal of the lesion/barrier, or by lesion bypass, which can require homologous recombination (HR). Fork restart can be aided by regression of stalled forks into chicken foot/Holliday Junction structures. HR repair involves recombination with the homologous sister chromatid to repair damaged DNA or restore replication forks. We have recently shown that stalled forks can be restarted in a Rad51-dependent pathway that does not involve recombination and may therefore be different from classical HR (5). Following prolonged replication blockages, forks can be processed into DNA double-strand breaks (DSBs), which are repaired by the classical HR pathway (5,6,7,8,9,10). We have recently shown that DSB repair by HR is not generally used as a method of replication restart following prolonged replication fork stalling and that firing of new origins is more likely to restart replication, followed by post-replicative HR repair of remaining DSBs (5).

There are several ssDNA binding proteins known to be involved in DNA repair processes (11), the best characterized of these is the Replication Protein A heterotrimer (RPA) (12,13). During the initiation of replication at origins of replication, DNA is unwound and RPA binds to the ssDNA produced, this stimulates the recruitment and activity of polymerase alpha at these sites (14). During elongation of the replication fork, RPA also functions to bind to the ssDNA generated by the progressing fork. In addition to its roles in unperturbed replication, RPA also has roles in the repair of various forms of DNA damage, including in the HR pathway (15). The RPA coated ssDNA is a substrate for the ATR:ATRIP complex, which binds to it and then initiates checkpoint signalling (16,17).

hSSB1, like RPA, is a ssDNA binding protein that is known to function in the repair of DNA damage (18,19). Unlike RPA however, hSSB1 is not required for normal S-phase progression (18). We and others have shown that hSSB1 is required for the recruitment of DNA repair factors to sites of DNA DSBs and stimulation of nucleases, such as Exonuclease 1 (Exo1) and the Mre11-Rad50-NBS1 (MRN) complex which resect the DNA to allow HR to proceed (20,21,22).

Given the large amounts of ssDNA generated in the repair of stalled/collapsed replication forks and the central role of hSSB1 in HR, we anticipated that hSSB1, in addition to RPA, could be involved in repair of stalled replication forks.

Here we demonstrate a role for hSSB1 in the repair of stalled and collapsed DNA replication forks. Specifically, depletion of hSSB1 renders cells sensitive to the replication fork-stalling agents hydroxyurea (HU) and camptothecin (CPT). In the absence of hSSB1, the crucial phosphorylation of the repair factor Chk1 is defective in response to stalled and collapsed replication forks. We also show that hSSB1 promotes the restart of stalled replication forks. Taken together, these data implicate hSSB1 in the repair of stalled and collapsed replication forks.

## MATERIALS AND METHODS

### Reagents, antibodies and cell lines

All cell lines were grown in Dulbecco's modified Eagle's medium supplemented with 10% Fetal Calf Serum. Antibodies used were as follows: mouse anti-γ-H2AX S139 (Milipore), rabbit anti-Rad51, goat anti-ATR and rabbit anti-Chk1 (Santa Cruz), rabbit anti-P-RPA S4/8 (Bethyl), mouse anti-RPA34, rabbit anti-γ-tubulin, mouse anti-α-tubulin, mouse anti-β-actin and rabbit anti-Mre11 (Sigma), rabbit anti-Chk1 S317, S345, S296, rat anti-RPA34 and rabbit anti-Histone H3 (Cell Signalling). Antibodies against hSSB1 were raised in sheep as described previously (18).

### Chemicals

Two micromolar HU was used for all experiments (unless otherwise stated).

### SiRNA and antisense RNA

SiRNA (esiRNA from Sigma or Stealth from Invitrogen) was transfected into HeLa cells using Lipofectamine 2000^TM^ (Invitrogen) as per manufacturer's instructions and samples were analysed 48 h after transfection. Antisense RNA was supplied by Gene Pharma and samples were analysed 24 h after transfection.

### DNA fibre analysis

MCF7 cells were transfected with 100 nM hSSB1 siRNA or control siRNA (Allstars negative control siRNA, Qiagen) using Lipofectamine 2000 according to manufacturer's instructions, and re-transfected with hSSB1 siRNA 24 h later. Twenty-four hours after the second transfection, cells were pulse labelled with 25 μM CldU for 20 min, washed three times with medium, incubated in 2 mM HU for 2 h, washed three times with medium and pulse labelled with 250 μM IdU for 1 h. Labelled cells were harvested and DNA fibre spreads prepared as previously described (23). CldU was detected by incubating acid treated fibre spreads with rat anti-BrdU monoclonal antibody (1:1000, AbD Serotec) for 1 h. Slides were fixed with 4% PFA (paraformaldehyde) and incubated with Alexa-Fluor 555-conjugated goat anti-rat IgG (1:500, Molecular Probes) for 1.5 h. IdU was detected using mouse anti-BrdU monoclonal antibody (1:1000, Becton Dickinson) over night at 4°C and Alexa-Fluor 488-conjugated goat anti-mouse IgG (1:500, Molecular Probes) for 1.5 h. Fibres were examined using a Biorad Radiance confocal microscope with a 60× oil immersion objective. For quantification of replication structures, at least 250 structures per experiment were counted using the ImageJ software (National Institutes of Health, http://rsbweb.nih.gov/ij/). Statistical analysis was performed using a one-tailed paired t-test.

### Immunoblotting

Immunoblotting was performed as described previously (11). Briefly, cells were lysed (lysis buffer: 20 mM Hepes Ph8, 150 mM KCl, 5% glycerol, 10 mM MgCl_2_, 0.5 mM EDTA, 0.02% NP-40, before use buffer was supplemented with NaF, NaVO_4_, PMSF and protease inhibitors) and sonicated. Lysates were cleared by centrifugation and protein concentrations were estimated using the standard Bradford assay (Bradford reagent supplied by Bio-Rad). Typically 50 μg of protein lysate was separated on a 4–12% sodium dodecyl sulphate-polyacrylamide gel electrophoresis gel (Invitrogen) and immunoblotted with the indicated antibodies. The immunoblots in Figure 3 were quantified using ImageJ software and normalized to total Chk1.

**Figure 1.**
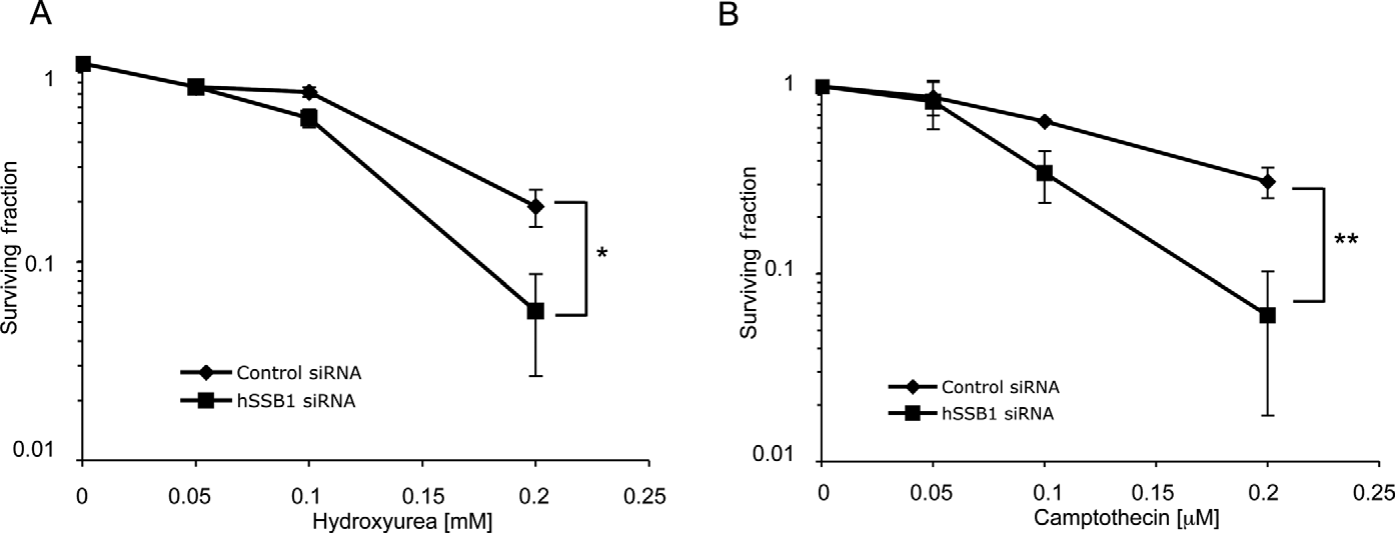
Sensitivity of hSSB1-deficient cells to replication stalling agents. HeLa cells were transfected with hSSB1 or control siRNA and treated with the indicated dose of the replication fork-stalling agents HU (**A**) or CPT (**B**). The means and s.d. (bars) of three independent experiments are shown. *P = 0.01. **P = 0.038.

**Figure 2.**
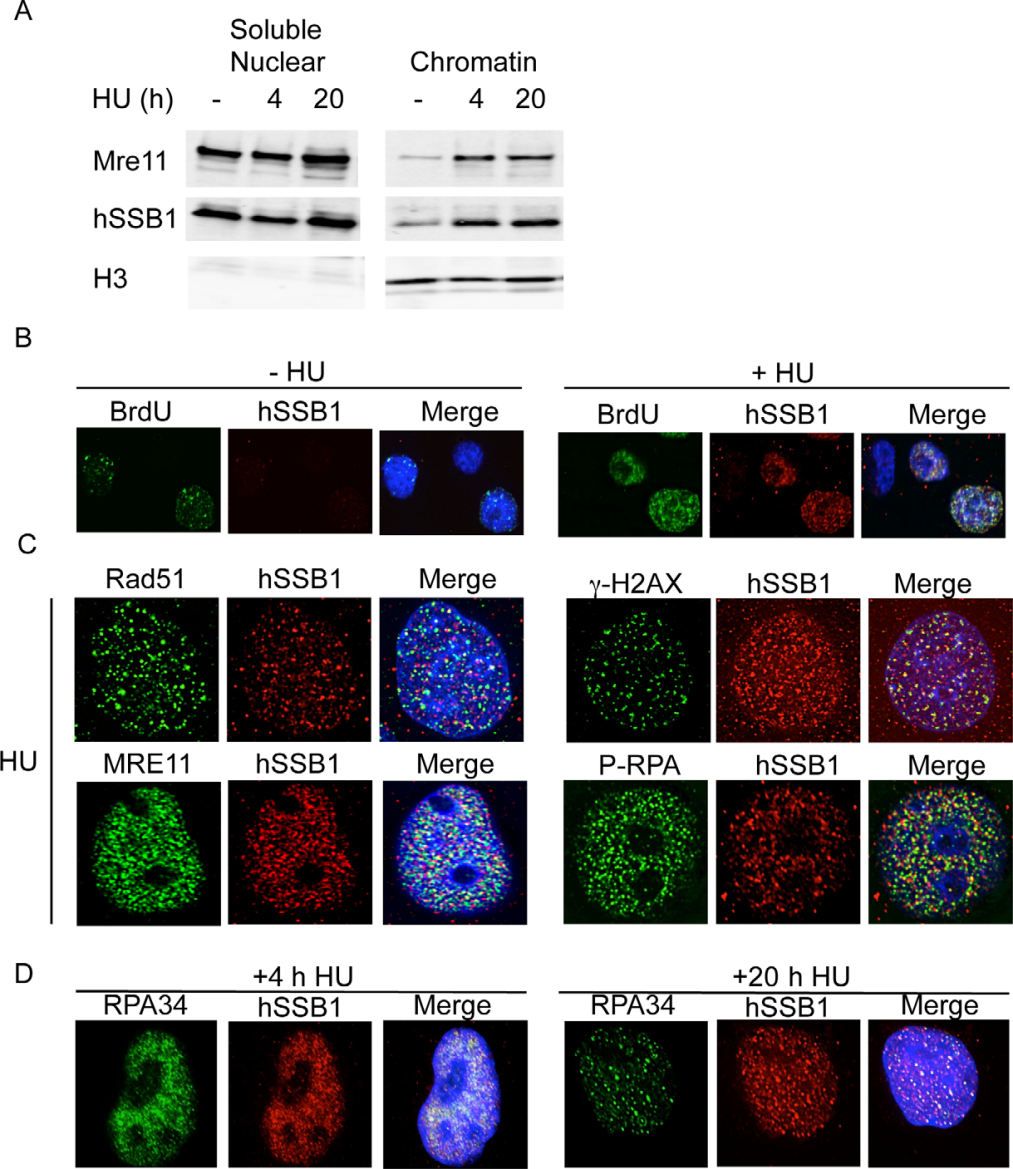
hSSB1 is localized to stalled replication forks. (**A**) hSSB1 is loaded onto chromatin following HU treatment. HeLa cells were treated or mock-treated with 2 mM HU for the indicated time before protein fractionations were carried out. (**B**) hSSB1 co-localizes with BrdU at stalled replication forks. HeLa cells were labelled with BrdU before treatment with 2 mM HU. Cells were then fixed and stained with the indicated antibodies. (**C**) hSSB1 partially co-localizes with other proteins required for repair of stalled replication forks. HeLa cells were treated with 2 mM HU for 20 h, fixed and stained with the indicated antibodies. (**D**) hSSB1 co-localizes with RPA34 after replication fork stalling. HeLa cells were treated with 2 mM HU for the indicated time and stained with the indicated antibodies.

**Figure 3.**
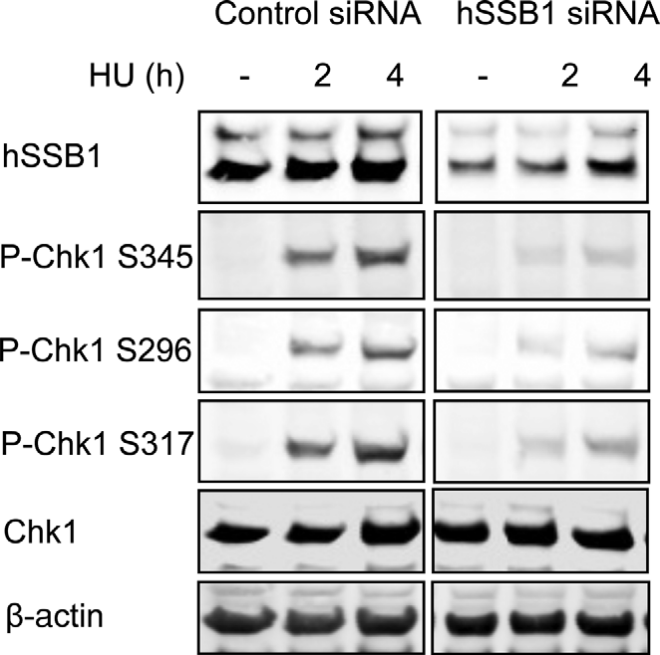
Defective DNA damage signalling in hSSB1-deficient cells. hSSB1 is required for Chk1 phosphorylation after replication fork stalling. HeLa cells were transfected with control or hSSB1 siRNA and treated or mock-treated with HU for the indicated time. Cell lysates were prepared and immunoblotted with the indicated antibodies.

### Colony forming assays

Colony forming assays were performed as described previously (24). Statistical analysis was performed using an unpaired t-test.

### Immunofluorescence

HeLa cells were seeded the day before siRNA transfection. Following siRNA transfection cells were allowed to grow for 48 h before treatment or mock-treatment with the indicated DNA damaging agent. After treatment cells were treated with an extraction buffer (25,26) for 10 min before fixation in 4% PFA. Cells were permeabilized with 0.2% Triton-X for 5 min and blocked in 3% bovine serum albumin for 30 min. Cells were incubated with indicated primary antibodies and Alexa-conjugated secondary antibodies for 1 h each at room temperature. Cells were stained with DAPI before mounting onto slides. Cells containing over 10 foci were scored as positive for foci unless otherwise stated. For the BrdU labelling (Figure 2A), cells were labelled with 10 mM BrdU for 20 min, prior to 30 min HU treatment and fixed in 4% PFA. Cells were incubated in 2M HCl for 30 min to denature the DNA. Cells were then permeabilized in 0.5% Triton-X. Cells were subjected to immunofluorescence with the indicated antibodies.

### Metaphase spreads

Two hundred ninety-three cells were treated with HU (2 mM) for 24 h. Cells were washed and grown for 3 h in fresh media. Colcemid was added and further incubated for 3 h. Metaphases were collected and analysed as described previously (27). For each treatment, 50 metaphases were analysed from three different experiments for each sample and mean aberrant metaphases were calculated. Statistical analysis was performed using an unpaired t-test.

### Comet assay

The neutral comet assay was employed to measure DSBs following HU treatment. HeLa cells were treated or mock-treated with HU (2 mM) for 20 h. Cells were embedded in agarose, lysed and subjected to electrophoresis, according to the Trevigen neutral comet assay protocol, with a few variations. The lysis buffer used contained: 2.5 M NaCl, 100 mM EDTA, 10 mM Tris (pH10), 1% Triton X-100. The electrophoresis was carried out with Tris/Borate/EDTA (TBE) buffer. Single cells were stained with Sybr Green I (Invitrogen) and at least 50 randomly selected cells per condition were analysed. The relative olive tail moment was measured using imageJ software. Statistical analysis was performed using an unpaired t-test.

### Subcellular fractionation

To detect binding of proteins to chromatin, subcellular fractionation was carried out as performed previously (26), according to the manufacturers instructions (Pierce). HeLa cells were treated with 2 mM HU for the indicated time, 48 h after transfection with control or hSSB1 siRNA.

## RESULTS

### hSSB1 is required for cell survival following replication fork damage

We have previously shown that hSSB1 is required for the repair of ionising radiation-induced damage (18). However, hSSB1-deficient human cells and hSSB1 knockout mouse bone-marrow cells show spontaneous generation of DNA DSBs that are most likely generated by the collapse of DNA replication forks and their incorrect repair (18,28). We have also shown previously that hSSB1 can bind to a replication fork-like structure *in vitro*, but is dispensable for normal S-phase progression in cycling cells (18). To determine if hSSB1 may also be involved in the repair of damaged DNA replication forks, we initially depleted HeLa cells of hSSB1 with siRNA and treated them with HU, which induces stalled and collapsed replication forks (via depletion of dNTP pools) (29). These cells demonstrated significant hypersensitivity to this agent at 0.2 mM (Figure 1A, Supplementary Figure S1). hSSB1-depleted cells were also significantly hypersensitive to CPT at 0.2 μM, an agent that also induces DSBs at replication forks by inhibiting Topisomerase II activity, causing it to remain cross linked to DNA (30) (Figure 1B), suggesting that hSSB1 is required for repair of damaged replication forks.

### hSSB1 is localized to chromatin following replication fork stalling and collapsing

Since the hypersensitivity of hSSB1-depleted cells to agents that damage replication forks suggests that hSSB1 may be required to repair DNA damage at replication forks, we next investigated whether hSSB1 was recruited to stalled replication forks. We have previously shown that treatment of cells with HU for over 12 h generates collapsed replication forks and subsequent DSBs (5). Therefore, to initially examine the role of hSSB1 at damaged replication forks we treated cells with HU for 4 h (to generate mostly stalled forks) or 20 h (to generate stalled and collapsed forks). hSSB1 was found to load onto chromatin in response to 4 h of HU treatment (Figure 2A). Since 4 h of HU treatment leads to replication fork stalling and not fork collapsing, this confirms that hSSB1 responds to replication fork stalling. Unsurprisingly, hSSB1 was also found to be recruited to chromatin after 20 h HU treatment. To confirm whether hSSB1 was recruited directly to sites of stalled replication forks, we labelled cells with BrdU to identify replication centres. hSSB1 was found to co-localize with BrdU after 30 min HU treatment, but not before, indicating that it is specifically recruited to stalled replication forks and is not located at replication forks during unperturbed replication (Figure 2B), supporting our previous observations that hSSB1 depletion had no effect on normal S-phase progression in cycling cells (18).

Prolonged treatments of HU lead to replication fork collapsing and results in DNA DSBs (5). Indeed, in cells treated with HU for 20 h, hSSB1 co-localized with other proteins known to be required to repair collapsed replication forks, including the phosphorylated form of H2AX (γH2AX) (Figure 2C). In addition to γH2AX, hSSB1 also partially co-localized with other proteins required for HR at collapsed forks, Rad51, phosphorylated RPA (p-RPA34 S4/8) and Mre11 (Figure 2C). hSSB1 has previously been shown to be in a complex with MRN of which Mre11 is a key component (20,21). Co-localization of faint hSSB1 and RPA34 could also be detected as early as 2 h of HU treatment (Supplementary Figure S2). hSSB1 was also found to localize to chromatin and co-localize with RPA34 from 4 to 20 h HU treatment, suggesting that it is present at both stalled and collapsed replication forks (Figure 2D and full time course presented in Supplementary Figure S3).

Recently, we placed hSSB1 in a complex with two other subunits INTS3 and C9ORF80 (31,32), thus it should also be noted that hSSB1 co-localized with C9ORF80 (Supplementary Figure S4) and INTS3 (Supplementary Figure S5) at collapsed replication forks.

### hSSB1 is required for the initiation of checkpoint signalling following stalled replication forks

We have previously shown that hSSB1 has an early role in the signalling and repair of DSBs and thus we next investigated whether hSSB1 played a similar early role in checkpoint activation at stalled replication forks. In response to replication fork stalling, the ATR kinase phosphorylates downstream effector proteins such as Chk1 to initiate checkpoint activation and repair. In response to DNA damage, ATR phosphorylates Chk1 at two main serine residues, S345 and S317, stimulating autophosphorylation at S296 (33,34). To determine if hSSB1 may function in this process, we next depleted cells of hSSB1 and examined phosphorylation of Chk1. Following 2 and 4 h HU treatment, which induces stalled replication forks, robust Chk1 S345, S317 and S296 phosphorylation was observed in control siRNA-transfected cells. In contrast, in hSSB1-depleted cells Chk1 phosphorylation was almost completely abrogated (Figure 3). This was further confirmed using another antisense RNA to deplete hSSB1 (Supplementary Figure S6). A small but significant, reproducible defect in Chk1 phosphorylation was also seen after 8–20 h HU treatment indicating that hSSB1 is required for ATR-dependent, maximal Chk1 phosphorylation following both replication fork stalling and collapsing (Supplementary Figure S7).

### hSSB1 is required for restart of stalled replication forks

**Figure 4.**
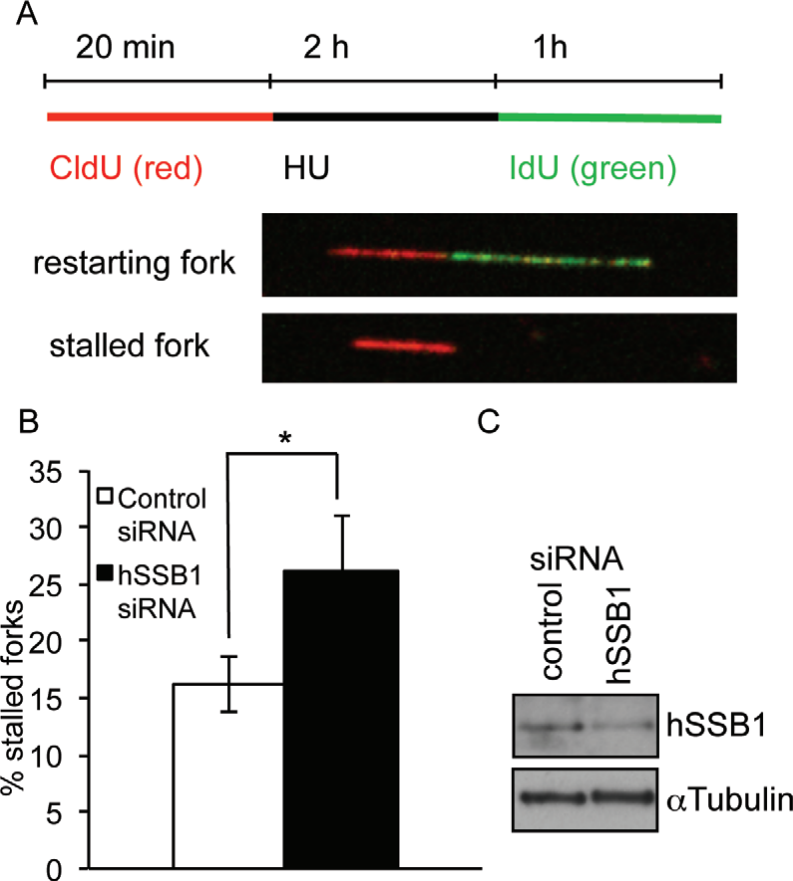
hSSB1 is required for replication restart of stalled replication forks. (**A**) Labelling protocols for DNA fibre analysis of replication forks. MCF7 cells were pulse labelled with CldU, treated with HU for 2 h, and released into IdU. (**B**) Fork restart in cells depleted of hSSB1. The means and standard error of the mean (bars) of four independent experiments are shown. *P = 0.05. (**C**) Knockdown of hSSB1.

Since hSSB1 is clearly required for the correct signalling of stalled replication forks and promotes cell survival after replication fork disruption, we next decided to examine the impact of hSSB1 depletion on the repair of stalled replication forks. In order to accurately examine replication fork restart, we next used a DNA fibre assay (35). Cells transfected with control or hSSB1 siRNA were pulse-labelled with CldU, forks stalled with 2 mM HU and released into media containing IdU (Figure 4A). We found that rather than globally slowing replication, a 60% depletion of hSSB1 leads to a small, but reproducible increase in the number of forks that do not resume replication after release from HU (Figure 4B and C, Supplementary Figures S7–S11 for more detailed analysis).

### Stabilisation of damaged replication forks is dependent upon hSSB1

**Figure 5.**
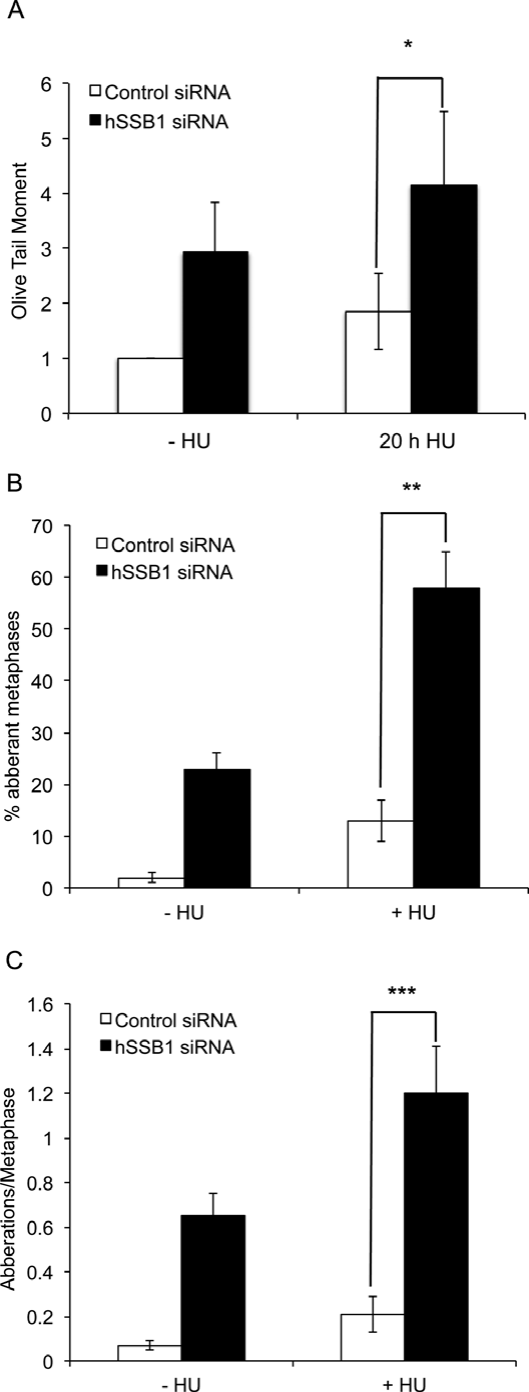
Genomic instability in hSSB1-depleted cells. (**A**) Comet assay showing the relative olive tail moment in hSSB1-deficient and control cells. HeLa cells were transfected with control or hSSB1 siRNA and were treated or mock-treated with 2 mM HU for 20 h and the neutral comet assay carried out. At least 50 cells were scored per condition and the results shown represent the mean and s.d. of three independent experiments. (**B**) Two hundred ninety-three cells were treated with HU (2 mM) for 24 h. Cells were washed and grown for 3 h in fresh media. Colcemid was added and further incubated for 3 h. For each treatment, 50 metaphases were analysed from three different experiments for each sample and mean aberrant metaphases were calculated. (**C**) Represents the number of aberrations/metaphase. Frequencies of spontaneous and HU-induced chromosome aberrations in control and hSSB1-deficient cells are indicated. Aberrations include mostly chromatid breaks, fragments, tri- and quadriradial figures. Fifty metaphases for each sample were analysed. The results shown represent the mean and s.d. of three independent experiments. *P = 0.034. **P = 0.0005. ***P = 0.0014.

Since we observe a defect in replication restart of stalled replication forks in the absence of hSSB1 (Figure 4) and given its role in the activation of ATR-dependent signalling (Figure 3), we next examined whether hSSB1 is required for replication fork stability during normal DNA replication and after a prolonged HU treatment (20 h). If forks are not stabilized they can collapse, leading to a DNA DSB. Using a neutral comet assay to measure DNA DSBs, we found that hSSB1-depleted cells had significantly more DNA DSBs even in the absence of fork-stalling agents (Figure 5A, Supplementary Figure S12). hSSB1-deficient cells treated with 2 mM HU for 20 h also showed significantly more DNA DSBs than HU-treated control siRNA transfected cells (Figure 5A). We also found that hSSB1-depleted cells displayed significantly more aberrant metaphases (Figure 5B) and an increased number of aberrations per metaphase (Figure 5C). In normally cycling, transformed cells, the predominant source of DNA DSBs breaks occurs in the S-phase of the cell cycle, suggesting that in the absence of hSSB1 DSBs may occur that cannot be repaired via HR. These findings demonstrate the importance of hSSB1 in maintaining and repairing replication forks and overall genomic stability.

### hSSB1 is required for recruitment of DNA repair components to sites of collapsed forks

**Figure 6.**
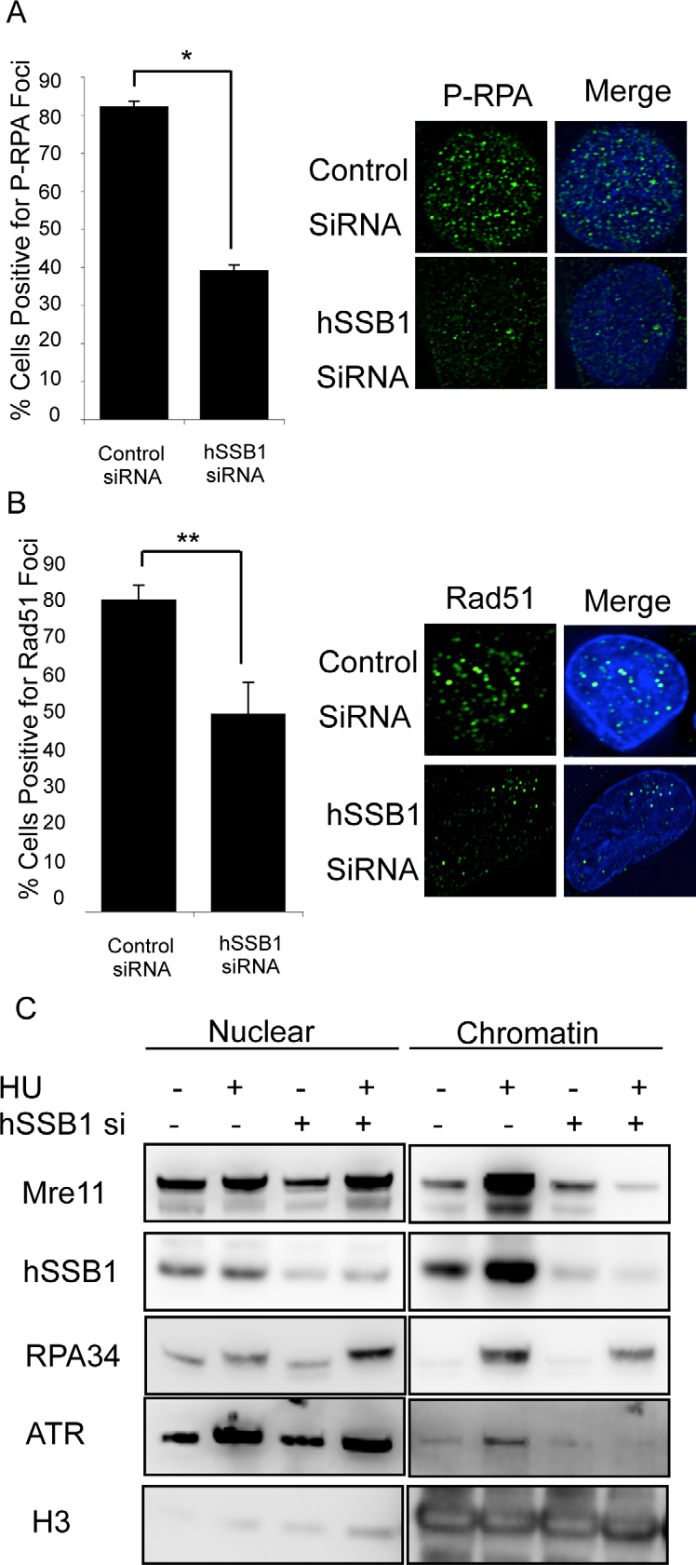
Defective recruitment of DNA repair proteins to collapsed replication forks in hSSB1-deficient cells. (**A**), (**B**) HeLa cells were transfected with control or hSSB1 siRNA and treated or mock-treated with 2 mM HU. Cells were fixed and stained with (A) p-RPA34 S4/8 or (B) Rad51. Cells were scored as being positive (>10 foci) or negative for foci. Representative images and the means and s.d. (bars) of three independent experiments are shown. (**C**) HeLa cells were transfected with mock or hSSB1 siRNA, treated with 2 mM HU and subcellular fractionations performed. Immunoblotting of the nuclear and chromatin fractions was carried out with the indicated antibodies. *P = 0.0043, **P = 0.032.

In the process of DNA repair of collapsed replication forks, many repair factors are recruited to the site of damage in a highly coordinated manner. To determine where hSSB1 may function in this process, we next depleted HeLa cells of hSSB1, mock-treated or treated cells with 2 mM HU for 20 h and carried out immunofluorescence. hSSB1-depleted cells showed significant defects in the phosphorylation of RPA, the recruitment of Rad51 and the nuclease Mre11 (Figure 6A and B, Supplementary Figure S13) to the damaged replication forks, suggesting that hSSB1 plays a crucial role in recruiting repair proteins to collapsed replication forks.

To explore this further, we performed subcellular fractionation to separate the cellular components. Following HU treatment, a number of DNA repair proteins can be observed to be loaded onto chromatin to perform roles such as maintaining replication fork stability and repairing the replication fork itself. We found that after 20 h HU treatment several proteins involved in DNA repair were loaded onto chromatin in control cells including Mre11, RPA34 and ATR as has previously been reported (36,37). In addition, hSSB1 was also loaded (Figure 6C) (36,37). In contrast, hSSB1-depleted cells displayed defective loading of Mre11 and ATR, suggesting that hSSB1 is required for the loading of DNA repair proteins onto chromatin after replication fork collapsing (Figure 6C). Loading of RPA34 was also reduced in hSSB1-deficient cells in response to prolonged HU treatment (Figure 6C). However, we were unable to detect a significant defect in RPA34 recruitment to foci in hSSB1-deficient cells in response to shorter treatments (4–8 h) (Supplementary Figure S14). We have previously demonstrated that hSSB1 interacts directly with the MRN complex through NBS1 and that this interaction is required for the recruitment and activity of the MRN complex at sites of DNA DSBs (20,21). Here, we demonstrate that in the absence of hSSB1, Mre11 is not recruited to damaged replication forks. Since Mre11 is required for stabilisation of replication forks and resection of DNA in order to complete HR, this suggests that hSSB1 is essential for replication fork repair following damage.

## DISCUSSION

There are a variety of mechanisms involved in the restoration of DNA replication after genotoxic stress. We have previously demonstrated a role for hSSB1 in HR repair of DNA DSBs (18). Here, we also highlight a role for hSSB1 in the repair of stalled and collapsed DNA replication forks. Our observations are supported by a recent publication that suggested hSSB1 is required to prevent accumulation of replication-associated DNA damage during skeletogenesis in mice (38).

The best characterized ssDNA binding protein involved in DNA replication in eukaryotes is the RPA heterotrimer, which is associated with the replication fork during normal replication (39). In contrast to RPA, we have shown here that hSSB1 associates with stalled/collapsed replication forks and cannot be detected at replication forks during unperturbed S-phase, suggesting a distinct role for hSSB1 in repair of replication forks. However, we cannot rule out that a small undetectable pool of hSSB1 may be associated with replication forks during S-phase to repair forks that stall or collapse, which would provide an explanation for the DNA damage that occurs in hSSB1-deficient cells in the absence of exogenous damage. We have previously shown that hSSB1 binds to DSBs and stalled replication fork-like structures (18), supporting this, here we show a role for hSSB1 at stalled and collapsed replication forks.

We have previously shown that hSSB1 is required to recruit RPA34 to sites of IR-induced DSBs and in the current study we found that maximal RPA34 accumulation and phosphorylation at collapsed replication forks was also dependent upon hSSB1. One key difference between Ionizing radiation (IR)-induced DSBs and HU-induced replication fork damage is that RPA is already present at replication forks in normally cycling cells. Some studies have shown that ssDNA coated with RPA can promote Chk1 phosphorylation (40), however others have shown that RPA is dispensable for Chk1 phosphorylation (41,42), this may suggest that another mechanism is responsible for the activation of ATR and subsequent Chk1 phosphorylation following replication fork damage. Here we show that recruitment of RPA to stalled replication forks is not sufficient to stimulate Chk1 phosphorylation in the absence of hSSB1. We suggest that this may point to a role for recruitment of hSSB1 as the mechanism required to stabilize replication forks and promote recruitment of DNA repair proteins, activate ATR and promote Chk1 phosphorylation following replication fork stalling and collapsing.

A key component of the MRN complex, Mre11, is required for resection of DNA ends (43). We have previously shown that hSSB1 is required for MRN-dependent resection of DNA DSBs (18,20,21) and a recent publication has shown that hSSB1 can stimulate the nuclease activity of Exo1 (44). Hence, it is easy to envisage that hSSB1 is also required for resection following replication fork collapse and indeed here we show that hSSB1 is also required for recruitment of Mre11 to collapsed replication forks (Figure 6, Supplementary Figure S13). Due to the defect in Mre11 recruitment, we propose that extensive resection is not occurring at collapsed replication forks in hSSB1-deficient cells. Chromatin fractionation showed that in the absence of hSSB1 less RPA was recruited to chromatin, suggesting that the accumulation and extension of RPA at collapsed replication forks was defective. This was also supported by the defect we observed in RPA34 phosphorylation at collapsed replication forks. RPA34 phosphorylation has been previously linked with resection (43,45) and the defective RPA phosphorylation seen in hSSB1-deficient cells supports our observation that Mre11 is not recruited and therefore the maximal resection required to recruit Rad51 and complete HR is not occurring in hSSB1-deficient cells.

Previously, we have shown that hSSB1 is required for ATM-dependent DNA damage signalling (18) and here, we also find a clear link between ATR and hSSB1. We have shown that hSSB1 is required for the loading of ATR onto chromatin (Figure 6) following replication fork damage. ATR is essential for restart of collapsed replication forks (37,46), and since this key protein is no longer recruited in hSSB1-depleted cells, it would also suggest a crucial role for hSSB1 in the repair of collapsed replication forks. We were unable to detect ATR loading at early times after HU (4 h) in control or hSSB1-deficient cells (data not shown), but given the defect in the phosphorylation of the key ATR substrate, Chk1 at this time point, it is likely that ATR activation in response to stalled-replication forks is disrupted in hSSB1-deficient cells.

Phosphorylation of Chk1 is a crucial stage of the response to damaged replication forks and has been shown to be required for activation and recruitment of Chk1 to chromatin following replication fork stalling (47). Moreover, we have previously shown that Chk1 is required to phosphorylate Rad51 to repair stalled/collapsed replication forks via HR and that this is dependent upon Chk1 S345 and S317 phosphorylation (48). Thus, we predicted that Rad51 function is also impaired in hSSB1-deficient cells.

DNA replication forks that are stalled for prolonged periods of time may collapse, leading to DSB formation, these are repaired via the Rad51-dependent HR pathway. We have previously proposed a role for hSSB1 in HR repair of DNA DSBs and here we also suggest that hSSB1 is involved in HR repair of collapsed replication forks. In support of this, we found that HU-induced Rad51 foci were significantly reduced in hSSB1-deficient cells. Reinforcing this notion, hSSB1-deficient cells accumulate spontaneous DNA damage (Figure 5 (18,28,38)) and it is likely that this damage is due to the collapse of replication forks in S-phase that are unable to be repaired via HR. This suggests that hSSB1 is required for replication fork stability after stalling and this is supported by our data showing that hSSB1-deficient cells accumulate DNA DSBs and chromosomal aberrations following replication fork stalling by HU (Figure 5). These data indicate that hSSB1 is either directly required to maintain replication fork stability following stalling or it functions to recruit other key factors that carry out this role. Indeed, we have shown that hSSB1 is required to recruit Mre11, which is known to be required to stabilize replication forks (49). In addition, ATR is also required to prevent chromosomal instability following replication fork stalling (43) and ATR is also not recruited in hSSB1-deficient cells.

Taken together, the data we present here confirms a role for hSSB1 in the restart of stalled replication forks. We propose that following replication fork stalling, hSSB1 has a role in promoting ATR activity, which in turn phosphorylates Chk1 and is required for replication restart via Rad51-dependent and HR-independent pathways (5). In addition, we also propose a role for hSSB1 in a model for repair of collapsed replication forks via HR, whereby hSSB1 is required for the stabilisation of damaged replication forks, promoting recruitment of Mre11. Mre11 then stabilizes collapsed replication forks which in turn allows recruitment of DNA repair proteins, promoting Rad51-dependent HR.

## SUPPLEMENTARY DATA

Supplementary Data are available at NAR Online.

## FUNDING

Australian Research Council [DP120103099, FT0991671 to D.J.R.]; National Health and Medical Research council [552472 to K.K.K.]; National Institutes of Health [CA129537, CA154320 to T.K.P.]. Source of Open Access funding: Australian Research Council [DP120103099 to D.J.R.]

*Conflict of interest*. None declared.

## Supplementary Material

SUPPLEMENTARY DATA

## References

[1] Huberman J.A., Riggs A.D. (1968). On the mechanism of DNA replication in mammalian chromosomes. J. Mol. Biol..

[2] Lomonosov M., Anand S., Sangrithi M., Davies R., Venkitaraman A.R. (2003). Stabilization of stalled DNA replication forks by the BRCA2 breast cancer susceptibility protein. Genes Dev..

[3] Walter J., Newport J. (2000). Initiation of eukaryotic DNA replication: origin unwinding and sequential chromatin association of Cdc45, RPA, and DNA polymerase alpha. Mol. Cell.

[4] Guo Z., Kumagai A., Wang S.X., Dunphy W.G. (2000). Requirement for Atr in phosphorylation of Chk1 and cell cycle regulation in response to DNA replication blocks and UV-damaged DNA in Xenopus egg extracts. Genes Dev..

[5] Petermann E., Orta M.L., Issaeva N., Schultz N., Helleday T. (2010). Hydroxyurea-stalled replication forks become progressively inactivated and require two different RAD51-mediated pathways for restart and repair. Mol. Cell.

[6] Carr A.M. (2002). DNA structure dependent checkpoints as regulators of DNA repair. DNA Repair (Amst).

[7] Carr A.M. (2002). Checking that replication breakdown is not terminal. Science.

[8] Cha R.S., Kleckner N. (2002). ATR homolog Mec1 promotes fork progression, thus averting breaks in replication slow zones. Science.

[9] Cobb J.A., Schleker T., Rojas V., Bjergbaek L., Tercero J.A., Gasser S.M. (2005). Replisome instability, fork collapse, and gross chromosomal rearrangements arise synergistically from Mec1 kinase and RecQ helicase mutations. Genes Dev..

[10] Sogo J.M., Lopes M., Foiani M. (2002). Fork reversal and ssDNA accumulation at stalled replication forks owing to checkpoint defects. Science.

[11] Skaar J.R., Richard D.J., Saraf A., Toschi A., Bolderson E., Florens L., Washburn M.P., Khanna K.K., Pagano M. (2009). INTS3 controls the hSSB1-mediated DNA damage response. J. Cell Biol..

[12] Oakley G.G., Patrick S.M. (2010). Replication protein A: directing traffic at the intersection of replication and repair. Front. Biosci..

[13] Vassin V.M., Anantha R.W., Sokolova E., Kanner S., Borowiec J.A. (2009). Human RPA phosphorylation by ATR stimulates DNA synthesis and prevents ssDNA accumulation during DNA-replication stress. J. Cell Sci..

[14] Tanaka T., Nasmyth K. (1998). Association of RPA with chromosomal replication origins requires an Mcm protein, and is regulated by Rad53, and cyclin- and Dbf4-dependent kinases. Embo J..

[15] Park M.S., Ludwig D.L., Stigger E., Lee S.H. (1996). Physical interaction between human RAD52 and RPA is required for homologous recombination in mammalian cells. J. Biol. Chem..

[16] Zou L., Elledge S.J. (2003). Sensing DNA damage through ATRIP recognition of RPA-ssDNA complexes. Science.

[17] Cortez D., Guntuku S., Qin J., Elledge S.J. (2001). ATR and ATRIP: partners in checkpoint signaling. Science.

[18] Richard D.J., Bolderson E., Cubeddu L., Wadsworth R.I., Savage K., Sharma G.G., Nicolette M.L., Tsvetanov S., McIlwraith M.J., Pandita R.K. (2008). Single-stranded DNA-binding protein hSSB1 is critical for genomic stability. Nature.

[19] Ashton N.W., Bolderson E., Cubeddu L., O’Byrne K.J., Richard D.J. (2013). Human single-stranded DNA binding proteins are essential for maintaining genomic stability. BMC Mol. Biol..

[20] Richard D.J., Cubeddu L., Urquhart A.J., Bain A., Bolderson E., Menon D., White M.F., Khanna K.K. (2011). hSSB1 interacts directly with the MRN complex stimulating its recruitment to DNA double-strand breaks and its endo-nuclease activity. Nucleic Acids Res..

[21] Richard D.J., Savage K., Bolderson E., Cubeddu L., So S., Ghita M., Chen D.J., White M.F., Richard K., Prise K.M. (2011). hSSB1 rapidly binds at the sites of DNA double-strand breaks and is required for the efficient recruitment of the MRN complex. Nucleic Acids Res..

[22] Yang S.H., Zhou R., Campbell J., Chen J., Ha T., Paull T.T. (2013). The SOSS1 single-stranded DNA binding complex promotes DNA end resection in concert with Exo1. Embo J..

[23] Henry-Mowatt J., Jackson D., Masson J.Y., Johnson P.A., Clements P.M., Benson F.E., Thompson L.H., Takeda S., West S.C., Caldecott K.W. (2003). XRCC3 and Rad51 modulate replication fork progression on damaged vertebrate chromosomes. Mol. Cell.

[24] Bolderson E., Savage K.I., Mahen R., Pisupati V., Graham M.E., Richard D.J., Robinson P.J., Venkitaraman A.R., Khanna K.K. (2012). Kruppel-associated Box (KRAB)-associated co-repressor (KAP-1) Ser-473 phosphorylation regulates heterochromatin protein 1beta (HP1-beta) mobilization and DNA repair in heterochromatin. J. Biol. Chem..

[25] Young D.B., Jonnalagadda J., Gatei M., Jans D.A., Meyn S., Khanna K.K. (2005). Identification of domains of ataxia-telangiectasia mutated required for nuclear localization and chromatin association. J. Biol. Chem..

[26] Bolderson E., Tomimatsu N., Richard D.J., Boucher D., Kumar R., Pandita T.K., Burma S., Khanna K.K. (2010). Phosphorylation of Exo1 modulates homologous recombination repair of DNA double-strand breaks. Nucleic Acids Res..

[27] Gupta A., Sharma G.G., Young C.S., Agarwal M., Smith E.R., Paull T.T., Lucchesi J.C., Khanna K.K., Ludwig T., Pandita T.K. (2005). Involvement of human MOF in ATM function. Mol. Cell Biol..

[28] Shi W., Bain A.L., Schwer B., Al-Ejeh F., Smith C., Wong L., Chai H., Miranda M.S., Ho U., Kawaguchi M. (2013). Essential developmental, genomic stability, and tumour suppressor functions of the mouse orthologue of hSSB1/NABP2. PLoS Genet..

[29] Bianchi V., Pontis E., Reichard P. (1986). Changes of deoxyribonucleoside triphosphate pools induced by hydroxyurea and their relation to DNA synthesis. J. Biol. Chem..

[30] Avemann K., Knippers R., Koller T., Sogo J.M. (1988). Camptothecin, a specific inhibitor of type I DNA topoisomerase, induces DNA breakage at replication forks. Mol. Cell Biol..

[31] Li Y., Bolderson E., Kumar R., Muniandy P.A., Xue Y., Richard D.J., Seidman M., Pandita T.K., Khanna K.K., Wang W. (2009). HSSB1 and hSSB2 form similar multiprotein complexes that participate in DNA damage response. J. Biol. Chem..

[32] Skaar J.R., Richard D.J., Saraf A., Toschi A., Bolderson E., Florens L., Washburn M.P., Khanna K.K., Pagano M. (2009). INTS3 controls the hSSB1-mediated DNA damage response. J. Cell Biol..

[33] Zhao H., Piwnica-Worms H. (2001). ATR-mediated checkpoint pathways regulate phosphorylation and activation of human Chk1. Mol. Cell Biol..

[34] Okita N., Minato S., Ohmi E., Tanuma S., Higami Y. (2012). DNA damage-induced CHK1 autophosphorylation at Ser296 is regulated by an intramolecular mechanism. FEBS Lett..

[35] Bryant H.E., Petermann E., Schultz N., Jemth A.S., Loseva O., Issaeva N., Johansson F., Fernandez S., McGlynn P., Helleday T. (2009). PARP is activated at stalled forks to mediate Mre11-dependent replication restart and recombination. Embo J..

[36] Fotedar R., Roberts J.M. (1992). Cell cycle regulated phosphorylation of RPA-32 occurs within the replication initiation complex. Embo J..

[37] Lupardus P.J., Byun T., Yee M.C., Hekmat-Nejad M., Cimprich K.A. (2002). A requirement for replication in activation of the ATR-dependent DNA damage checkpoint. Genes Dev..

[38] Feldhahn N., Ferretti E., Robbiani D.F., Callen E., Deroubaix S., Selleri L., Nussenzweig A., Nussenzweig M.C. (2012). The hSSB1 orthologue Obfc2b is essential for skeletogenesis but dispensable for the DNA damage response in vivo. Embo J..

[39] Dimitrova D.S., Gilbert D.M. (2000). Stability and nuclear distribution of mammalian replication protein A heterotrimeric complex. Exp. Cell Res..

[40] Choi J.-H., Lindsey-Boltz L.A., Sancar A. (2007). Reconstitution of a human ATR-mediated checkpoint response to damaged DNA. Proc Natl Acad Sci U.S.A..

[41] Recolin B., Van der Laan S., Maiorano D. (2012). Role of replication protein A as sensor in activation of the S-phase checkpoint in Xenopus egg extracts. Nucleic Acids Res..

[42] Vidal-Eychenie S., Decaillet C., Basbous J., Constantinou A. (2013). DNA structure-specific priming of ATR activation by DNA-PKcs. J. Cell Biol..

[43] Sartori A.A., Lukas C., Coates J., Mistrik M., Fu S., Bartek J., Baer R., Lukas J., Jackson S.P. (2007). Human CtIP promotes DNA end resection. Nature.

[44] Jiang G., Plo I., Wang T., Rahman M., Cho J.H., Yang E., Lopez B.S., Xia F. (2013). BRCA1-Ku80 protein interaction enhances end-joining fidelity of chromosomal double-strand breaks in the G1 phase of the cell cycle. J. Biol. Chem..

[45] Sirbu B.M., Couch F.B., Feigerle J.T., Bhaskara S., Hiebert S.W., Cortez D. (2011). Analysis of protein dynamics at active, stalled, and collapsed replication forks. Genes Dev..

[46] Wang X., Zou L., Lu T., Bao S., Hurov K.E., Hittelman W.N., Elledge S.J., Li L. (2006). Rad17 phosphorylation is required for claspin recruitment and Chk1 activation in response to replication stress. Mol. Cell.

[47] Jiang K., Pereira E., Maxfield M., Russell B., Goudelock D.M., Sanchez Y. (2003). Regulation of Chk1 includes chromatin association and 14–3–3 binding following phosphorylation on Ser-345. J. Biol. Chem..

[48] Sorensen C.S., Hansen L.T., Dziegielewski J., Syljuasen R.G., Lundin C., Bartek J., Helleday T. (2005). The cell-cycle checkpoint kinase Chk1 is required for mammalian homologous recombination repair. Nat. Cell Biol..

[49] Trenz K., Smith E., Smith S., Costanzo V. (2006). ATM and ATR promote Mre11 dependent restart of collapsed replication forks and prevent accumulation of DNA breaks. Embo J..

