# Supplementary material for: Human single-stranded DNA binding protein 1 (hSSB1/NABP2) is required for the stability and repair of stalled replication forks
Source: Nucleic Acids Res. 2014 May 5;42(10):6326–36. doi: 10.1093/nar/gku276 (PMC4041449; doi:10.1093/nar/gku276)
Supplement: SUPPLEMENTARY DATA [file supp_42_10_6326__index.html]

Human single-stranded DNA binding protein 1 (hSSB1/NABP2) is required for the stability and repair of stalled replication forks — Human single-stranded DNA binding protein 1 (hSSB1/NABP2) is required for the stability and repair of stalled replication forks — SUPPLEMENTARY DATA 

# Human single-stranded DNA binding protein 1 (hSSB1/NABP2) is required for the stability and repair of stalled replication forks

## SUPPLEMENTARY DATA

**Files in this Data Supplement:**

- SUPPLEMENTARY DATA
