# Supplementary material for: Human single-stranded DNA binding protein 1 (hSSB1/NABP2) is required for the stability and repair of stalled replication forks
Source: Nucleic Acids Res. 2014 May 5;42(10):6326–36. doi: 10.1093/nar/gku276 (PMC4041449; doi:10.1093/nar/gku276)
Supplement: SUPPLEMENTARY DATA [file supp_gku276_nar-02529-h-2013-File008.pdf]

S1

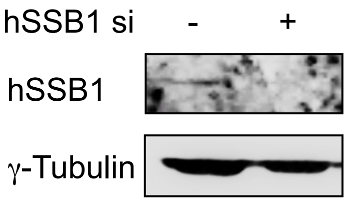

S1. HeLa cells were transfected with control or hSSB1 siRNA and cell lysates were prepared after 48 hours. Immunoblotting was performed with the indicated antibodies.

S2

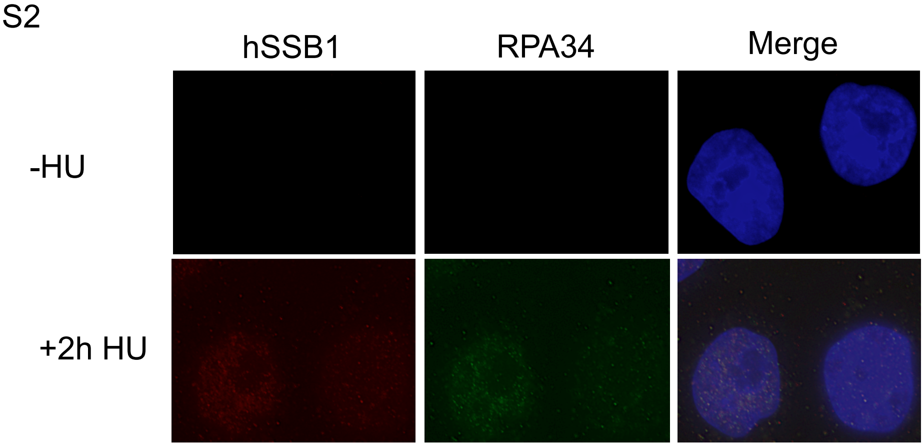

S2. hSSB1 co-localises with RPA34 at stalled replication forks. HeLa cells were treated with 2mM HU for 2 hours, pre-extracted, fixed and stained with the indicated antibodies. Faint hSSB1 and RPA34 foci are detected at stalled replication forks as early as 2 hours after HU treatment.

S3

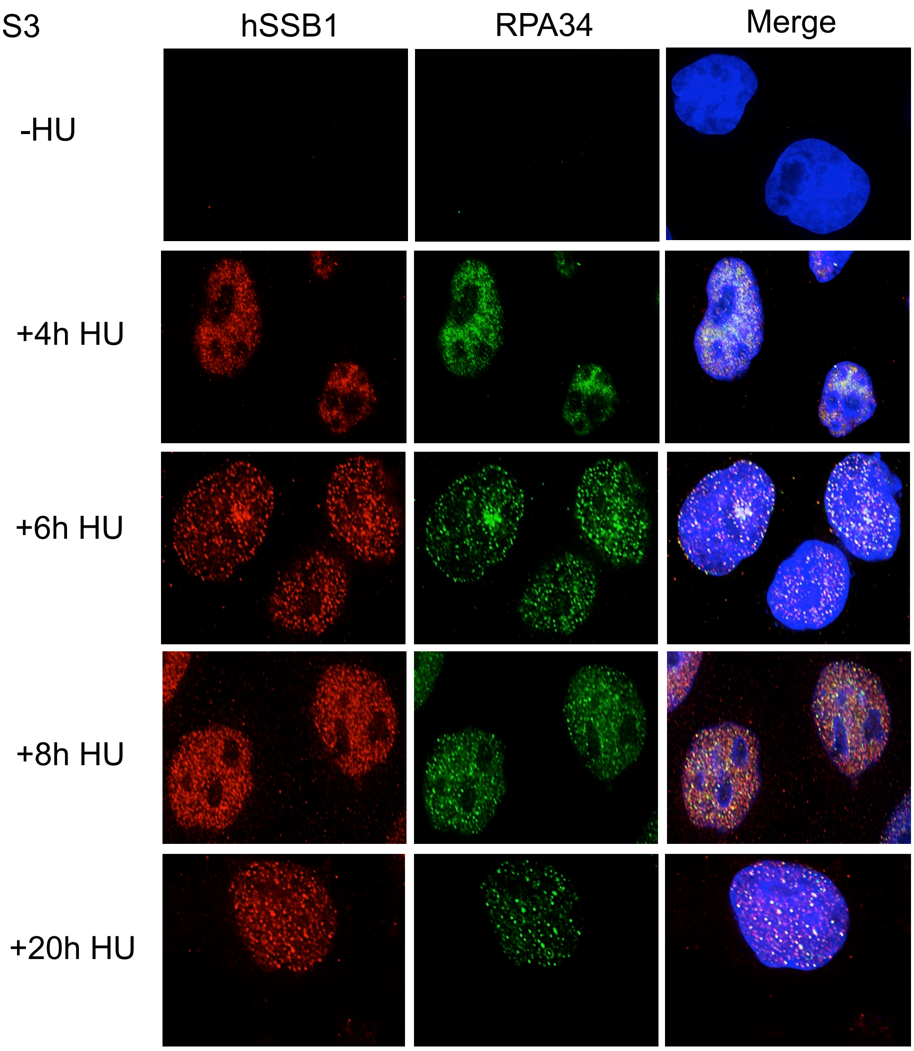

S3. hSSB1 co-localises with RPA34 at stalled replication forks. HeLa cells were treated with 2mM HU for the indicated time, pre-extracted, fixed and stained with the indicated antibodies.

S4

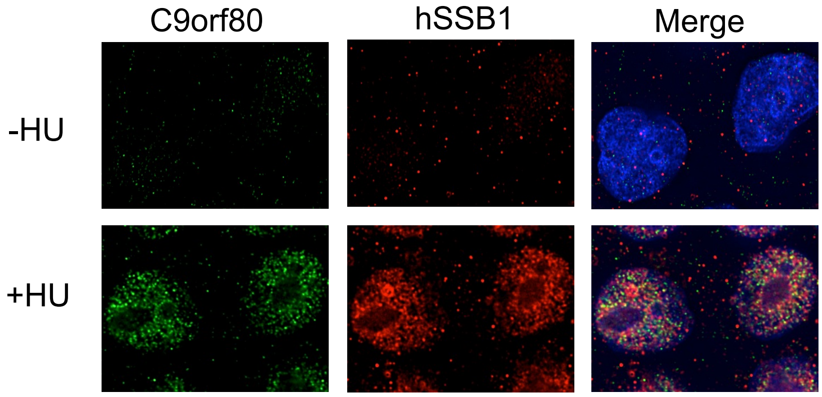

S4. hSSB1 co-localises with C9orf80 at collapsed replication forks. HeLa cells were treated with 2mM HU for 24 h, pre-extracted, fixed and stained with the indicated antibodies.

S5

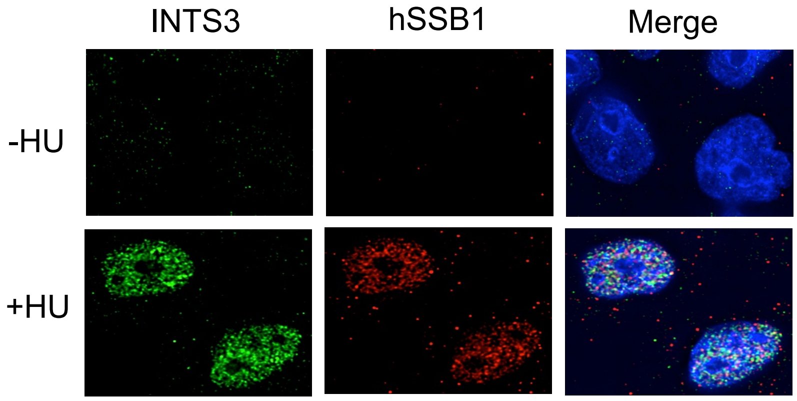

S5. hSSB1 co-localises with Ints3 at collapsed replication forks. HeLa cells were treated with 2mM HU for 24 h, pre-extracted, fixed and stained with the indicated antibodies.

S6

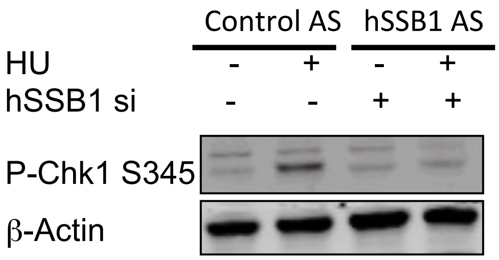

S6. U2OS cells were transfected with antisense (AS) RNA targeting control or hSSB1. After 24 hours cells were treated or mock-treated with 2mM HU for 4h and cell lysates were prepared. Immunoblotting was performed with the indicated antibodies.

S7

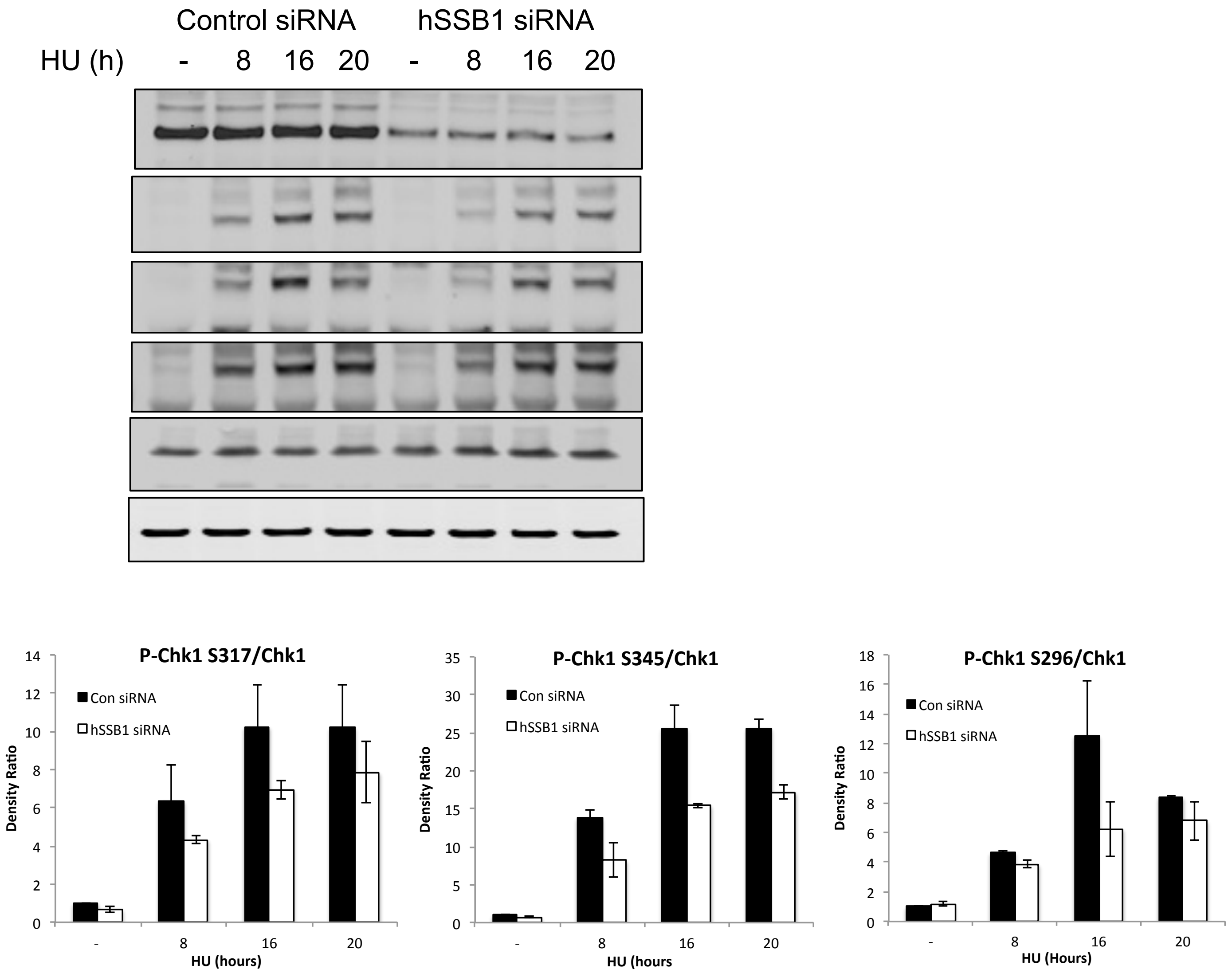

S7. hSSB1 is required for Chk1 phosphorylation following prolonged HU treatment. HeLa cells were transfected with control or hSSB1 siRNA and treated or mock-treated with HU for the indicated time. Cell lysates were prepared and immunoblotted with the indicated antibodies. The p-Chk1 immunoblots were quantified using Image J software and the density ratio is presented. The means and s.e.m. from 2 independent experiments are shown.

S8

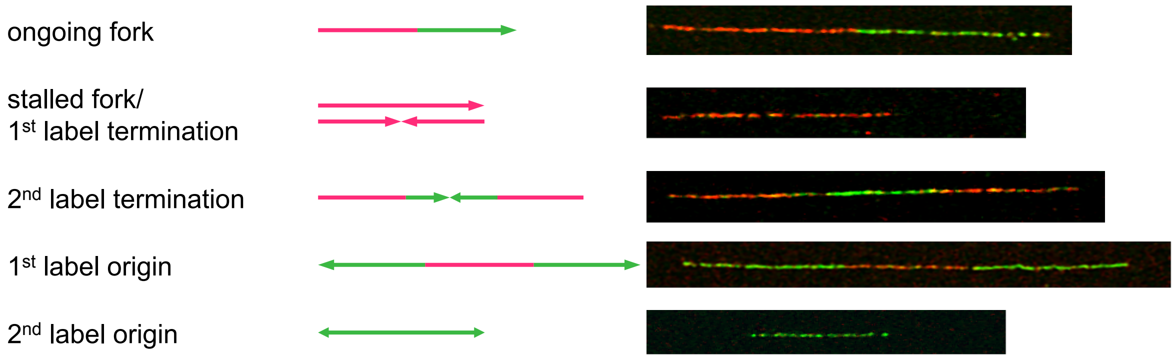

S8. Schematics and example images of replication structures quantified.

S9

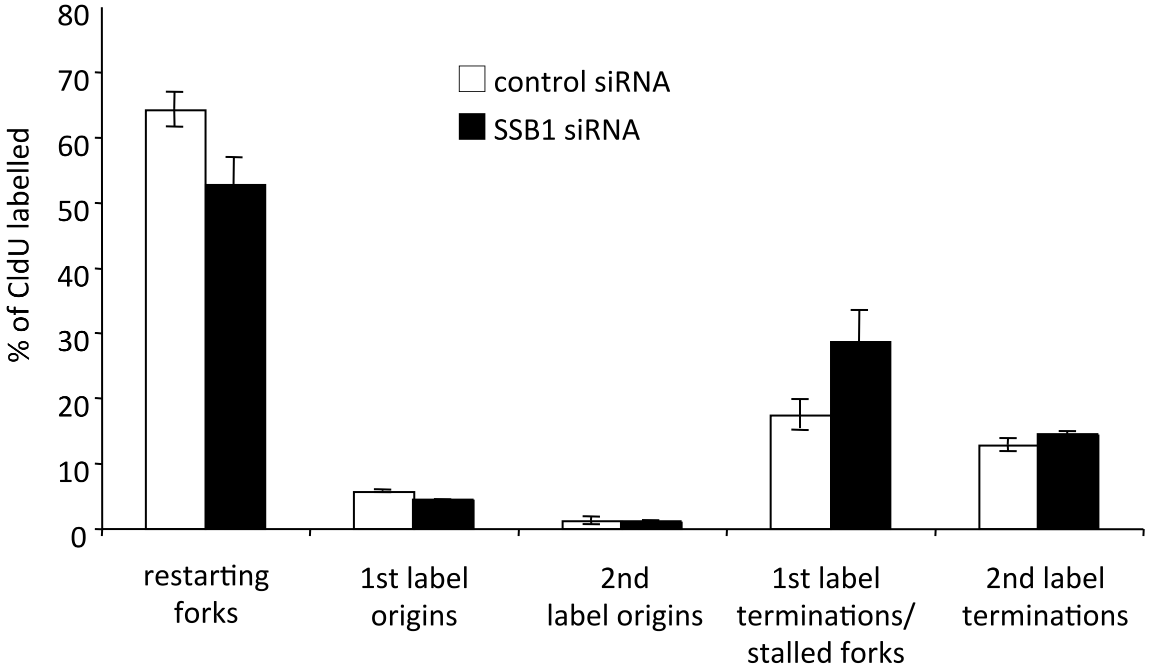

S9. Quantification of all replication structures in cells as in Figure 4A-C. Replication structures are shown as percentage of all CldU-labelled tracks. The means and S.E.M. (bars) of three independent experiments are shown.

S10

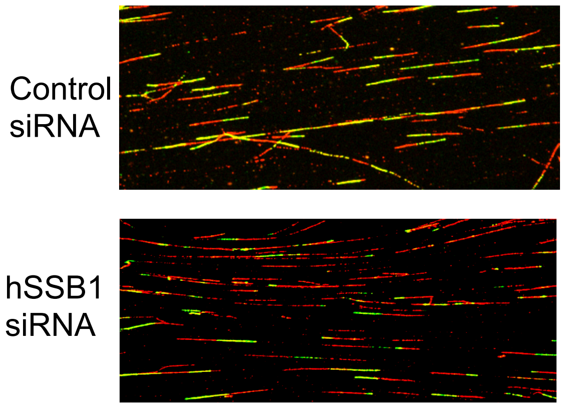

S10. Representative images of DNA fibre tracks in control and hSSB1 siRNA transfected cells.

S11

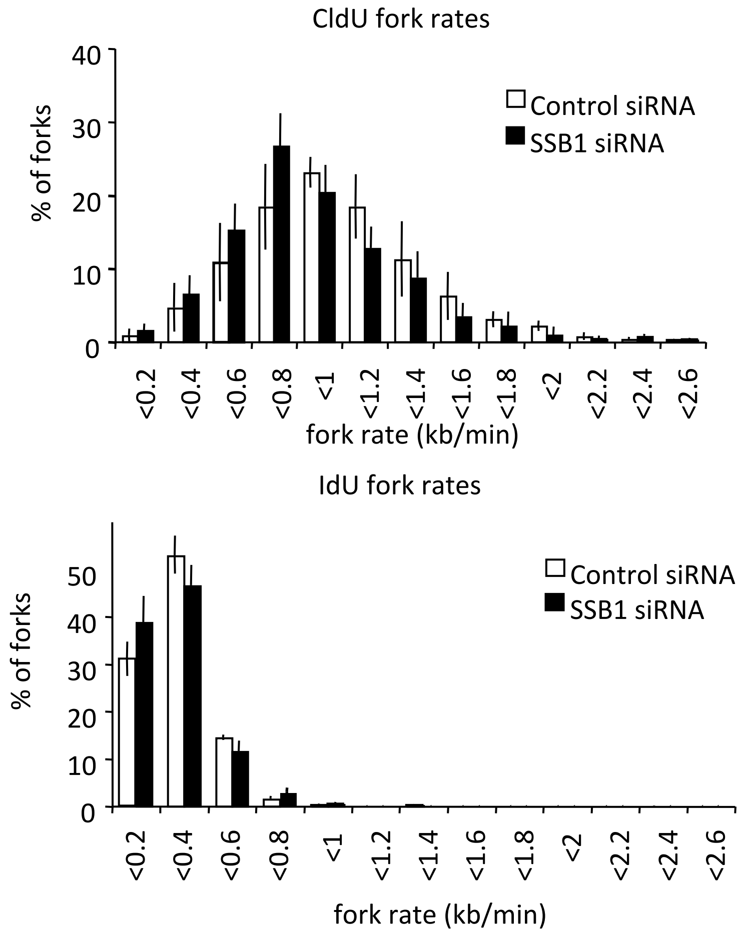

S11. CldU and IdU fork rates in control and hSSB1 siRNA transfected cells.

S12

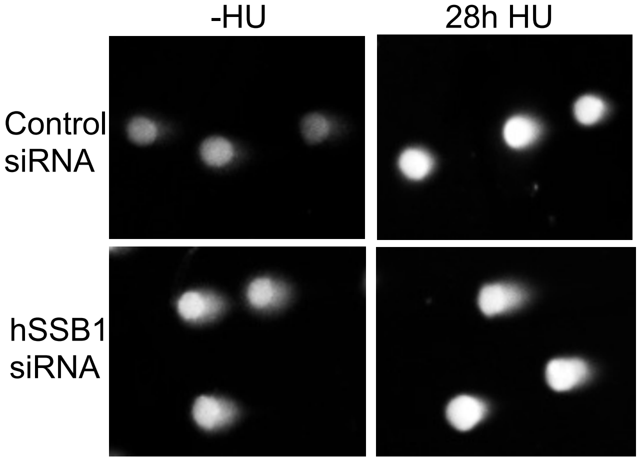

S12. Representative comet assay images.

S13

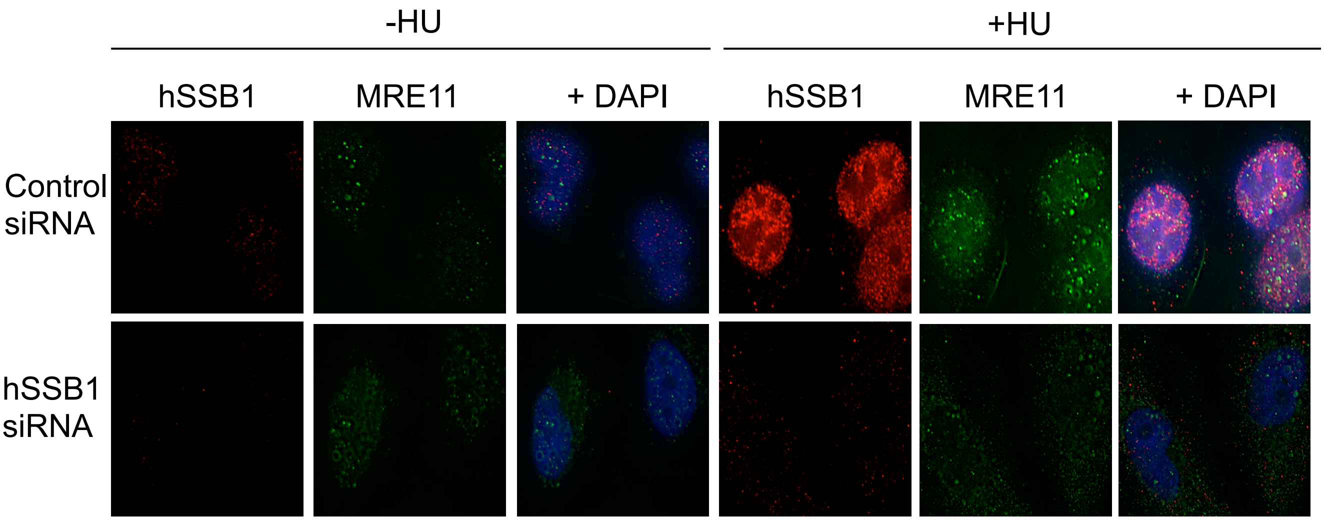

S13. hSSB1 is required for recruitment of Mre11 to collapsed replication forks. HeLa cells were transfected with hSSB1 or control siRNA, treated with 2mM HU for 24 h, pre-extracted, fixed and stained with the indicated antibodies.

S14

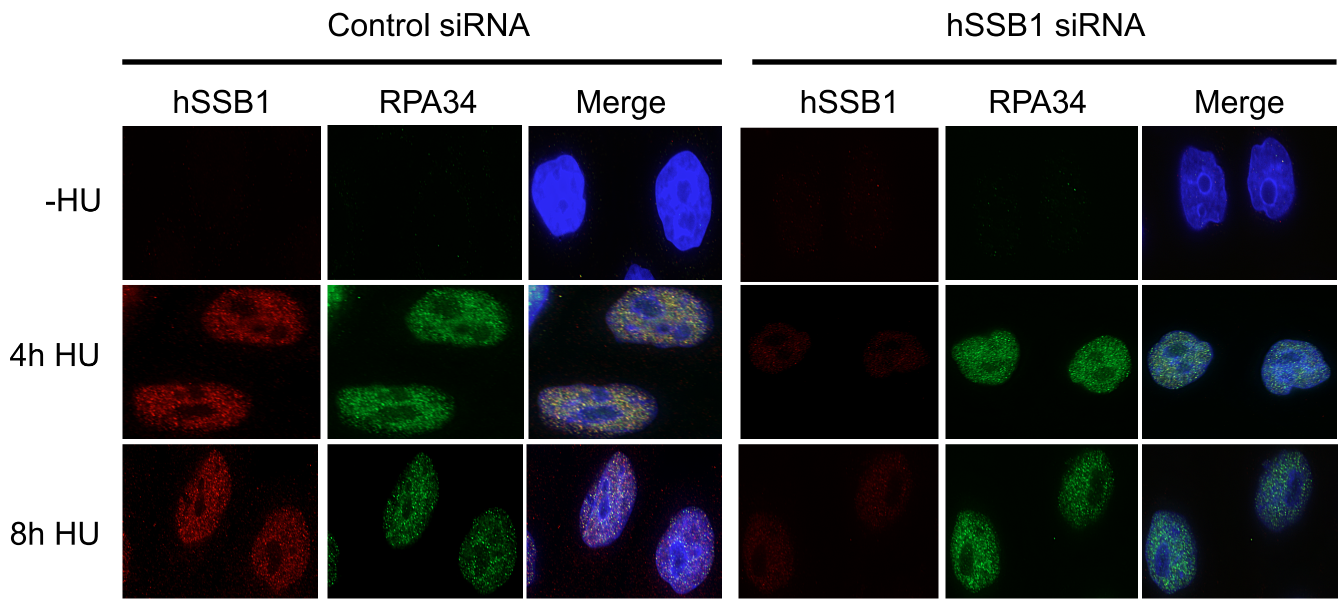

S14. hSSB1 is not required for recruitment of RPA34 to stalled replication forks. HeLa cells were transfected with hSSB1 or control siRNA, treated with 2mM HU for the indicated time, pre-extracted, fixed and stained with the indicated antibodies.
